# A Positive Quadriceps Active Test, without the Quadriceps Being Active

**DOI:** 10.1155/2019/6135632

**Published:** 2019-12-27

**Authors:** D. C. Kieser, E. Savage, P. Sharplin

**Affiliations:** Department of Orthopaedic Surgery and Musculoskeletal Medicine, University of Otago, Christchurch, New Zealand

## Abstract

**Case:**

A 55-year-old male with a chronic isolated grade 3 PCL injury who demonstrates a positive quadriceps active test without activating his quadriceps musculature.

**Conclusion:**

Gravity and hamstring contraction posteriorly translate the tibia into a subluxed position. Subsequent gastrocnemius contraction with the knee flexed causes an anterior tibial translation by virtue of the mass enlargement of the gastrocnemius muscular bulk, the string of a bow effect, and the anterior origin of the gastrocnemius in relation to the posterior border of the subluxed tibia aided by the normal posterior tibial slope.

## 1. Introduction

Posterior cruciate ligament (PCL) injuries have been classically described as having a posterior station, posterior draw test, and a positive dial test at 90 degrees of knee flexion [1–4]. In addition, the quadriceps' active test has been used to further define this injury [4, 5]. This test was first described by Daniel and colleagues in 1988 and is performed by having the knee at 90 degrees flexion and getting the patient to extend the knee against resistance [6]. During this action, the activation of the quadriceps musculature is believed to anteriorly translate the tibia from a posteriorly subluxed position to a reduced neutral position. The translation is clinically observed and used to further confirm the diagnosis, with a reported sensitivity of 54-98% and a specificity of 97-100% [2, 6].

However, when assessing the patients during this type of examination, one can often observe that some patients do not activate the quadriceps musculature, yet they successfully reduce their knee joint. In these patients, gravity or hamstrings activation posteriorly subluxes the tibia. Then, almost paradoxically, it appears that these patients contract their gastrocnemius to anteriorly translate their tibias. This anterior translation is then described as a positive quadriceps active test, despite the quadriceps not being active.

This finding may be important in the patient with quadriceps dysfunction where the clinical diagnosis of a PCL injury is difficult. In addition, typical rehabilitation for PCL injuries include quadriceps strengthening, but not all patients benefit equally. In these patients, gastrocnemius muscle function may offer additional advantage.

We present a case of a patient with a positive quadriceps activation test achieved without activating the quadriceps.

## 2. Case Report

Both verbal and written informed consents were obtained from the patient for this case report.

**Figure 1:**
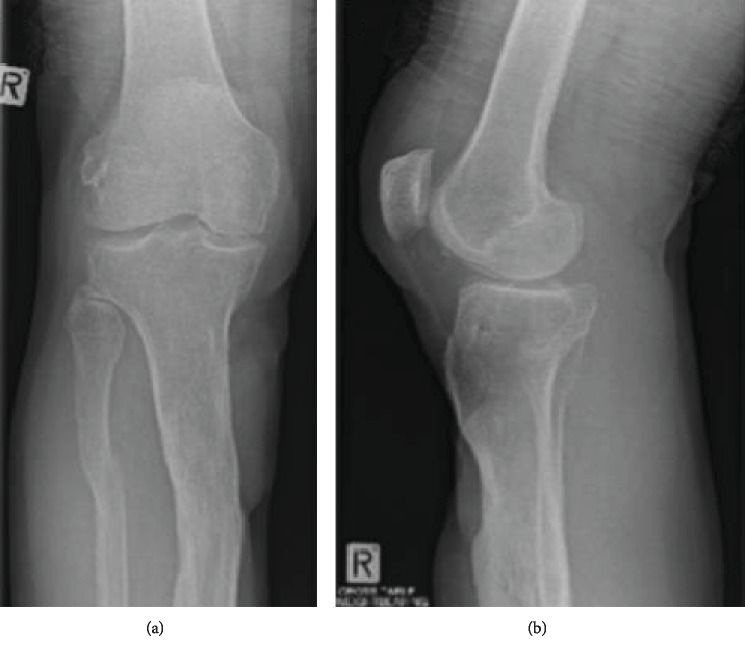
Anteroposterior and lateral radiographs showing the healed proximal tibia fracture and posterior subluxation of the tibia. Weight-bearing AP (a) and lateral (b) radiographs of MR AD showing evidence of a healed proximal tibia and fibula fracture and posterior subluxation of the tibia on the femur. Importantly, the posterior slope of his proximal tibia is within normal limits measuring 6 degrees.

Mr. AD is a 55-year-old male truck driver who was involved in a motorbike accident at the age of 17 years, where he sustained a right proximal tibia and grade 3 PCL injury. He was managed conservatively for his PCL injury and denied any residual significant symptoms despite his continued clinical PCL laxity. He presented to the orthopaedic service for another reason, when incidentally, we reviewed his knee as a teaching case. During his examination, he was found to have anterior proximal tibial scarring, an S-shaped coronal tibial deformity but normal overall mechanical axis and normal tibial alignment in the sagittal plane. He had a positive posterior sag with a grade 3 posterior station, a positive posterior draw, reverse Lachmans, and dial test at 90 degrees of flexion but normal at 30 degrees. He was stable in the varus/valgus plane. His X-rays confirmed a healed proximal tibia and fibula fracture as well as posterior subluxation of his tibia on his femur (Figure 1). Hence, his clinical and radiographic signs confirmed an isolated grade 3 PCL injury in the presence of a previous proximal tibia and fibula fracture.

**Figure 2:**
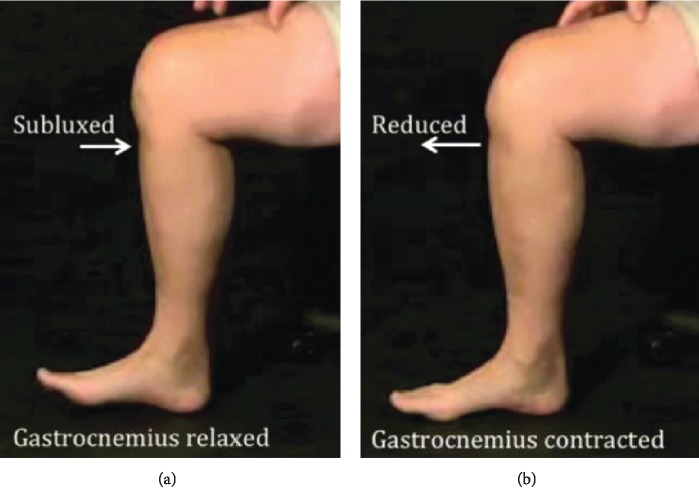
Clinical photographs with the gastrocnemius relaxed (a) and contracted (b). Note that the photograph is taken in the seated position to better illustrate the position of the foot. In (a), the ankle is dorsiflexed to illustrate calf relaxation, whereas in (b), the ankle is plantarflexed to illustrate gastrocnemius contraction. Patient with a positive “quads active test” who does not activate his quads to reduce his tibiofemoral articulation. Note that in (a), his gastrocnemius is relaxed (hence his dorsiflexed ankle) and his tibia is posteriorly subluxed, but in (b), his gastrocnemius is contracted and the tibiofemoral articulation is reduced. These findings were consistent when lying supine or sitting erect (as above).

On performing a quadriceps active test at 90 degrees of knee flexion, it was noted that he did not activate his quadriceps but rather contracted his calf musculature to reduce his knee joint (Figure 2).

## 3. Discussion

The lateral gastrocnemius muscle originates on the lateral surface of the lateral femoral condyle, while the medial head arises from the posterior surface of the medial femoral condyle and the popliteal surface of the distal femur [7]. The two heads are surrounded by the deep fascia and converge side-by-side before inserting into the dense aponeurosis of the tendo-achilles [7].

**Figure 3:**
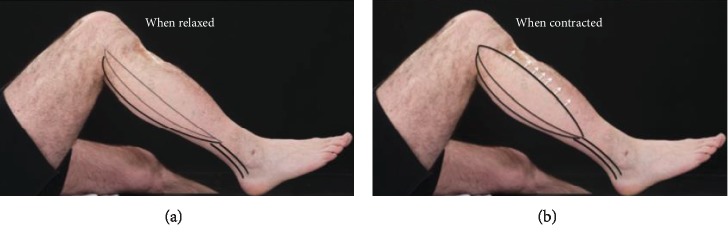
Clinical photographs with the effect of gastrocnemius contraction illustrated. Patient with a PCL injury and posterior sag sign. Diagrammatic depiction of the gastrocnemius muscle bulk while relaxed (a) and contracted (b) to demonstrate the shortening but widening of the gastrocnemius muscle bulk with contraction.

The majority of the muscle mass lies at the level of the proximal tibia. Therefore, when the gastrocnemius contracts, the muscle shortens and enlarges in the axial plane, pressing on the posterior soft tissues and the posteriorly subluxed tibia, causing an anterior force which reduces the knee joint (Figure 3).

**Figure 4:**
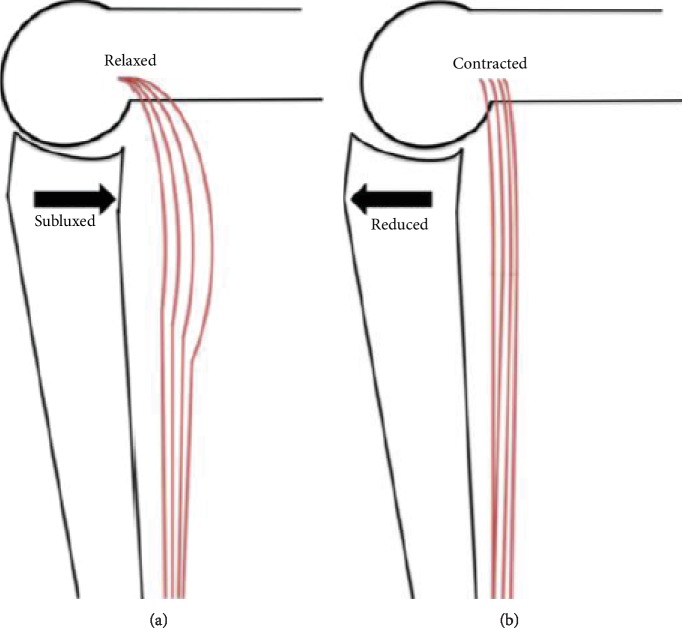
Illustration of gastrocnemius contraction reducing the posterior subluxation. Diagrammatic depiction of the knee joint showing posterior subluxation of the tibia with the gastrocnemius relaxed (a) and anterior translation to a reduced position with the gastrocnemius contracted (b). This translation is driven by the gastrocnemius acting like the string of a bow, aided by the normal posterior slope of the tibia and origin of the gastrocnemius being anterior to the posterior border of the subluxed tibia.

In addition, with contraction of the gastrocnemius on a fixed ankle and knee, the musculotendinous structure acts as the string of a bow forcing the posteriorly subluxed tibia anteriorly, aided by the natural posterior slope of the proximal tibia and the origin of the gastrocnemius being anterior to the posterior border of the subluxed tibia (Figure 4).

## 4. Conclusion

We propose that in some patients, a positive quadriceps active test can occur without quadriceps activation. We propose that gravity or hamstring contraction posteriorly translates the tibia into a subluxed position. Subsequent gastrocnemius contraction with the knee flexed causes an anterior tibial translation by virtue of the mass enlargement of the gastrocnemius muscular bulk, the string of a bow effect, and the anterior origin of the gastrocnemius in relation to the posterior border of the subluxed tibia aided by the normal posterior tibial slope. This finding aids the clinical diagnosis even in patients with quadriceps dysfunction and suggests that gastrocnemius strengthening may enhance rehabilitation after PCL injuries.

## References

[1] Loos W. C., Fox J. M., Blazina M. E., Del Pizzo W., Friedman M. J. (1981). Acute posterior cruciate ligament injuries. *American Journal of Sports Medicine*.

[2] Rubinstein R. A., Shelbourne K. D., McCarroll J. R., VanMeter C. D., Rettig A. C. (1994). The accuracy of the clinical examination in the setting of posterior cruciate ligament injuries. *American Journal of Sports Medicine*.

[3] Malanga G. A., Andrus S., Nadler S. F., McLean J. (2003). Physical examination of the knee: a review of the original test description and scientific validity of common orthopedic tests. *Archives of Physical Medicine and Rehabilitation*.

[4] The R. B. (2005). Orthopaedic physical examination.

[5] Jung Y. B., Lee E. W., Koo B. H. (1998). Reliability of the manual maximal displacement test and quadriceps active test with KT-1000 TM arthrometer in the assessment of posterior instability of the knee. *Journal of Korean Knee Society*.

[6] Daniel D. M., Stone M. L., Barnett P., Sachs R. (1988). Use of the quadriceps active test to diagnose posterior cruciate ligament disruption and measure posterior laxity of the knee. *The Journal of Bone & Joint Surgery*.

[7] McMinn R. M. H. (2005). *Last’s Anatomy*.

